# *ADIPOQ* Gene Variants Associated with Susceptibility to Obesity and Low Serum Adiponectin Levels in Healthy Koreans

**DOI:** 10.4178/epih/e2011003

**Published:** 2011-04-25

**Authors:** Ji Wan Park, Jungyong Park, Sun Ha Jee

**Affiliations:** 1 Department of Medical Genetics, College of Medicine, Hallym University, Chuncheon, Korea.; 2 Institute for Health Promotion, Graduate School of Public Health, Yonsei University, Seoul, Korea.; 3 Department of Epidemiology and Health Promotion, Graduate School of Public Health, Yonsei University, Seoul, Korea.; 4 Metabolic Syndrome Research Initiatives, Seoul, Korea.

**Keywords:** Adiponectin, Single nucleotide polymorphism, Obesity, Body mass index

## Abstract

**OBJECTIVES:**

This study aimed to measure the association between the adiponectin, C1Q and collagen domain-containing (*ADIPOQ*) gene variants and obesity in Koreans.

**METHODS:**

Three single nucleotide polymorphisms located in the *ADIPOQ* gene were genotyped in a population-based cross-sectional study of 986 healthy Koreans. Three different case-control groups (i.e. G1, G2, and G3) were defined according to body mass index (BMI) and serum adiponectin levels. Allelic and genotypic associations of this gene with obesity were measured using multivariate logistic regression analyses in each group.

**RESULTS:**

The G allele of -11377C>G, a polymorphism located in the promoter region of the *ADIPOQ* gene (odds ratio (OR), 1.48; 95% confidence interval, 1.13-1.94) and most haplotypes including this allele significantly increased the risk for obesity. However, the OR decreased from 3.98 (G1 group) to 2.90 (G2 group) and 2.30 (G3 group) when a less strict definition of obesity was used. Most haplotypes, including this allele, significantly increased the risk of obesity. The statistical evidence from the GG genotype of -11377C>G (OR, 3.98) and the GT/GT diplotype composed of -11377G>C and +45T>G (OR, 5.20) confirmed the contribution of the G allele toward a predisposition for obesity.

**CONCLUSION:**

These results suggest the contribution of the *ADIPOQ* gene toward susceptibility to obesity in healthy Koreans. The high-risk genotypes and haplotypes identified here may provide more information for identifying individuals who are at risk of obesity.

## INTRODUCTION

Obesity is a prevalent condition and a modifiable risk factor for various complex diseases, including diabetes and cardiovascular disease. Although the current World Health Organization (WHO) definitions for being overweight and obesity are body mass index (BMI) of ≥25-29.9 kg/m^2^ and ≥30 kg/m^2^, respectively, different BMI cut-off points of 23-24.9 kg/m^2^ and ≥25 kg/m^2^, respectively, were suggested for the Asian population [1,2]. In Korea, the prevalence of obesity (BMI ≥25 kg/m^2^) has dramatically increased over the past decade and the third Korea National Health & Nutrition Examination Survey reported that the overall prevalence of obesity in Korean adults was 30.6% (29.4% in women and 32.4% in men) in 2001 [3].

Adiponectin decreases body weight by increasing lipid oxidation in muscles and other organs such as the pancreas and liver [4]. The concentration of adiponectin in plasma was shown to be negatively correlated with BMI [5]. In humans, adiponectin is encoded by portions of exons 2 and 3 among the three exons of the adiponectin, C1Q and collagen domain-containing (*ADIPOQ*) gene (also denoted as *APM1* or *ACDC*) located on chromosome 3q27 [6]. The 13 single nucleotide polymorphisms (SNPs) in the *ADIPOQ* gene were previously reported in Japanese and French populations [7]. Two SNPs located on exon 2 (+45T>G, a synonymous mutation, Gly15Gly) and intron 2 (+276G>T) of the *ADIPOQ* gene were reported to be associated with both plasma adiponectin concentrations and type 2 diabetes (T2D) in Japanese, German, and Italian populations [7-9]. The G allele of the SNP -11377C>G located in the promoter region was also shown to be associated with lower adiponectin levels and severe obesity among Danish women [10]. Neither the mechanism responsible for controlling the synthesis of adiponectin nor the regulation of *ADIPOQ* gene expression have been fully determined as yet. The effect of the *ADIPOQ* gene on the risk of obesity may vary according to ethnicity, age, and he degree of obesity across populations [11].

Thus, this study aimed to perform an analysis of the association between the *ADIPOQ* gene and BMI, an index of obesity, whilst considering plasma adiponectin levels in a Korean population. We defined three groups of two extremes for comparison according to a combination of BMI levels and plasma adiponectin concentrations after adjusting for age and sex in 986 healthy Koreans.

## MATERIALS AND METHODS

### Study subjects

The study population consisted of 10,169 subjects who had participated in routine health examinations at the Health Promotion Center, Yonsei University Severance Hospital during the period of April 2006 to July 2008. The analyses excluded subjects with any disease, including diabetics (with fasting serum glucose ≥126 mg/dL), and those with prior usage of lipid-lowering drugs, as well as all participants with missing information on BMI or adiponectin levels. Finally, 986 subjects, aged 21 to 81 yr old, were genotyped for subsequent analyses. The Institutional Review Board of Human Research of Yonsei University approved the study, and written informed consent was obtained from all subjects before participation.

### Data collection

Each participant was interviewed using a structured questionnaire to collect histories of cigarette smoking (non-smoker, ex-smoker, or current smoker) and alcohol consumption (non-drinker or any alcohol drinker), as well as other demographic characteristics such as age and gender. The weight and height of each participant were measured in light clothing. The BMI was calculated as weight (kg) divided by height squared (m^2^).

### Measurement of biomarkers

For the clinical chemistry assay, serum was separated from peripheral venous blood samples obtained from each participant after 12 hr of fasting and stored at -70℃. Biomarkers for metabolic syndrome, such as fasting blood glucose, were measured using a Hitachi-7600 analyzer (Hitachi Ltd., Tokyo, Japan). The adiponectin level was measured using an enzyme-linked immunosorbent assay (ELISA; B-Bridge International Inc., Sunnyvale, CA, USA). Data quality control was performed in accordance with the procedures of the Korean Association of Laboratory Quality Control.

### Genotyping of SNPs

Genomic DNA was isolated from lymphocytes by using a DNA isolation kit according to the protocol of the manufacturer (WIZARD Genomic DNA purification kit; Promega Corp., Madison, WI, USA). In several populations other than Korean, three SNPs in the *ADIPOQ* gene were found to be associated with the levels of adiponectin and obesity [11]. Thus, the SNPs -11377C>G (rs266729), +45T>G (rs2241766), and +276G>T (rs1501299) were genotyped using the SNP-IT™ (SNP-Identification Technology) assay with the SNPstream 25K® System (Orchid Biosciences, Princeton, NJ, USA), as previously described [12]. The development of blue and/or yellow colors corresponding to two alleles was analyzed with an ELISA reader and the final genotype calling was made by the QC Review™ program (Orchid Biosciences). The genotyping success rate was an average of 99.12% and four duplicates of control DNA on each plate showed a 99% consistency rate for the genotype calling.

### Statistical analysis

**Figure 1:**
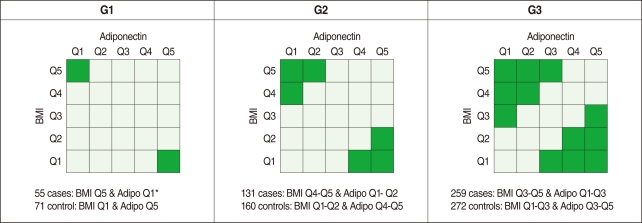
Case and control definitions by both body mass index (BMI) and plasma adiponectin concentration. Q, stands for quintile.

Analysis of variance (ANOVA) was used to evaluate whether these three SNPs were associated with plasma adiponectin concentration and BMI. Variations in these SNPs were significantly associated with adiponectin level and BMI (data not shown). Although obesity was not defined according to the WHO definition (i.e. a BMI of 25 kg/m^2^ or higher for Asians) in this study, obesity is known to positively correlate with BMI and reversely correlate with the level of adiponectin, and the *ADIPOQ* gene is the gene of interest as a susceptibility gene of obesity [13]. Therefore, we divided our study subjects into five groups (quintiles) of BMI (<22.7, 22.7-24.1, 24.2-25.4, 25.5-26.9, and ≥27.0 kg/m^2^ for men and <21.2, 21.2-22.8, 22.9-24.0, 24.1-25.5, and ≥25.6 kg/m^2^ for women) and five groups of adiponectin (<3.7, 3.8-4.7, 4.8-6.0, 6.1-7.9, and ≥8.0 µg/mL for men and <5.6, 5.6-7.1, 7.2-9.1, 9.2-12.1, and ≥12.2 µg/mL for women), respectively. We then defined three different comparison groups according to both the level of BMI and the plasma adiponectin concentration to compare obese individuals versus slim individuals, as shown in Figure 1 (i.e. G1, G2, and G3).

The minor allele frequency (MAF) and a chi-square test for the Hardy-Weinberg equilibrium (HWE) at each SNP, and the pair-wise linkage disequilibrium (LD) by both *D*' and *r*^2^ were computed among the controls of the G3 group using the Haploview program [14]. In order to measure the risk of obesity for alleles and genotypes of individual SNPs, as well as for haplotypes and diplotypes composed of two or three SNPs, multiple logistic regression models were tested in each of the three comparison groups after adjusting for age and other potential confounding factors such as gender, smoking status, and alcohol consumption. Genotypic odds ratios (GORs) and 95% confidence intervals (95% CIs) for heterozygotes and homozygotes were calculated separately, and then the best genetic model for each marker was determined using the Stata version 9.0 (StataCorp, College Station, TX, USA). To determine whether an insignificant result observed in the genotypic test was caused by a type II error, the power was computed using the web browser 'Genetic Power Calculator' [15].

## RESULTS

As shown in Table 1, 73.1% of the study population was composed of men (with a mean age of 49.5 yr old), and 26.9% of the population was female (mean: 51.2 yr old). The average BMI values were 24.8 and 23.6 kg/m^2^ for men and women, respectively. The plasma adiponectin level was approximately 30% higher in women (9.1 µg/mL) than in men (6.2 µg/mL). No gender difference was observed in the homeostasis model assessment of insulin resistance (HOMA-IR) levels. As shown Figure 1, the numbers of cases and controls increased from G1 (55 cases, 71 controls) to G3 (259 cases, 272 controls).

The major alleles for each marker of -11377C>G, +45T>G, and +276G>T in the controls of the G3 group were C, T, and G, respectively. All three markers showed sufficient heterozygosity, and no evidence of deviation from HWE was observed in any marker (Table 2). Three SNPs were not found to be in strong linkage disequilibrium based on the values of *r*^2^. Although individuals with the GG genotype of -11377C>G had a higher BMI and lower adiponectin concentration (25.1 kg/m^2^ and 6.7 µg/mL) compared to individuals with the CC reference genotype (24.4 kg/m^2^ and 7.1 µg/mL), the difference was not statistically significant (p=0.09) (Table 3).

### Allelic and genotypic odds ratios for individual SNPs

As shown in Table 4, the G allele and the GG genotype of the -11377C>G SNP significantly increased the risk of obesity (OR, 1.48 and 2.30, respectively, in the G3 group), although the regression model for the genotypic test was marginally significant (p=0.06). However, the OR decreased as the number of subjects increased (i.e. when the less strict definition of obesity was used) from 3.98 (group G1) to 2.90 (group G2) and 2.30 (group G3). The GG homozygotes of +45T>G showed a significantly low OR of 0.10, but only one case and eleven controls were available in the G1 group. The TT homozygotes of +276G>T were found to increase the risk of obesity, but this finding was not statistically significant. A recessive mode of inheritance was the best-fit to our data for all three markers in the most extreme comparison group of obesity (i.e. the G1 group) (data not shown). To test whether or not the number of subjects was sufficient to detect the effect size for each genotype, we modeled our study power under a recessive mode of inheritance. The G3 group composed of 259 cases and 272 controls achieved 80% power to significantly detect the OR over 2.29 and 2.06 under the assumption of disease prevalence, 30%; and disease allele frequencies, 0.24 and 0.28, respectively. In the current study, the *ADIPOQ* gene variants did not show a consistent trend of increasing or decreasing obesity for age. For instance, the ORs of the -11377C>G GG genotype were 2.4, 4, and 1.34 for the case-control groups less than 45 yr old (composed of 91 cases and 91 controls), 45-54 yr old (98 cases and 100 controls), and greater than 55 yr of age (83 cases and 68 controls), respectively, in the case of the G3 group.

### Haplotype and diplotype odds ratios for two or three SNPs

As shown in Table 5, the GT haplotype composed of the risk alleles of both SNPs -11377C>G and +45T>G significantly increased the risk of obesity in the G3 group (OR, 1.65; 95% CI, 1.17-2.32). Although the OR for the GG haplotype was significantly high (OR=4.02), the number of subjects was not sufficient. In Model 2, the CT and GG haplotypes consisted of one risky allele of either -11377C>G or +276G>T that significantly increased the risk of obesity (i.e. OR, 2.87 and 3, respectively, in the G1 group), whereas the GT haplotype consisted of two risky alleles that moderately increased the risk of obesity only in the G3 group (OR, 1.52; 95% CI, 1.03-2.25). In Model 3, the risk of obesity among those with the GTG haplotype was three-fold higher compared to the reference group in G1, whereas it decreased to 1.87 (1.20-2.91) in the G3 group. With two and three markers there were three and seven marker combinations, respectively (i.e. 2^n^-1) for which a haplotype-based test could be carried out. Even without considering the LD between the markers, the multiple comparison levels of significance for haplotypes consisting of two and three SNPs were 0.017 and 7×10^-3^, respectively. The GT (p=0.004), GG (p=0.006) and GTG haplotypes (p=0.005) in each model for the G3 group were significant even after the Bonferroni correction. The GG haplotype in Model 2 also yielded a Bonferroni level of significance in both G1 (p=0.003) and G2 groups (p=0.013).

The increased risk as the number of risky alleles increased in diplotypes of two SNPs, -11377C>G and +45T>G (i.e. OR, 2.96, 3.54, and 5.20 for CG/CT, CT/GT, and GT/GT, respectively), confirmed the contributions of the G and T alleles of each SNP in predisposing toward obesity (table not shown). This result also showed that the mutant alleles of two SNPs, -11377C>G and +276G>T, increased the risk of obesity whereas the G allele of +45T>G was protective.

## DISCUSSION

In the present study, three SNPs located on the *ADIPOQ* gene, which have previously been studied for an association with obesity in other ethnic groups [16], were evaluated in healthy Koreans. The C, T, and G alleles were the major alleles at SNPs -11377C>G, +45T>G, and +276G>T, respectively, as shown in other studies, including non-diabetic Japanese and non-diabetic Koreans [7,17]. A common allele, C, of -11377C>G was strongly associated with a lower plasma adiponectin level, a higher BMI, or T2D in some studies [18,19], whereas a rare allele, G, was associated with the same phenotypes in other studies [10,20]. Similar trends toward inconsistent results across studies were observed for the +45T>G and +276G>T loci. The rare G allele at the +45T>G locus significantly increased the risk of obesity in non-diabetic Germans and in Japanese with T2D [7,8], while Loos et al. [21] recently reported that carriers of the rare GG homozygote showed a leaner phenotype than carriers of the common T allele. The G allele at the +276G>T locus was associated with lower plasma adiponectin concentrations and higher insulin resistance only in subjects with a high BMI (≥26.7) in non-diabetic Korean and Japanese populations [7,17]. Homozygotes for the haplotype T45-G276 had a higher body weight and waist circumference and lower plasma adiponectin concentrations in non-diabetic Italians and non-diabetic obese Koreans [17,22,23].

In our study, the G allele of SNP -11377C>G (OR, 1.48; 95% CI, 1.13-1.94) and the T allele at the +45T>G locus were identified as being risk alleles for obesity, even after issues of study power and multiple testing were taken into account, where obesity was defined using both a higher BMI and a lower adiponectin concentration compared to the reference group. The T allele of the +276G>T locus was not statistically significant, but it increased the risk of obesity. Possible explanations for these inconsistent results across the studies include not only differences in population characteristics (e.g. demographic, genetic, and environmental factors, diagnostic criteria, etc.) but also the possibility that the configurations of genotypes or hapolotypes were mixed up in some studies. Minor allele frequencies of each SNP were different even from Japanese HapMap data (e.g. 28.9% of Japanese HapMap data vs. 24% of data in the current study for the SNP -11377C>G). Therefore, more prospective studies of a better design are necessary to confirm the effects of each variant on the development of obesity [17,24]. As shown in other Korean studies, linkage disequilibrium was not observed between SNPs -11377C>G, +45T>G or +276G>T, whereas two SNPs, +45T>G and +276G>T were in imperfect (*r*^2^=0.14) but complete linkage disequilibrium (*D*'=1) in our study [18,23].

Overall, a stricter definition of the phenotype increased the statistical power to detect evidence of an association in our study. Although significantly high ORs were observed in the CG haplotype of -11377G>C and +45T>G (OR, 4.02) and the GGT haplotype (OR, 11.52), these high ORs may have been the result of the relatively small sample size (Table 4). Our results replicated the reverse relationship between BMI and plasma adiponectin (Table 3) and the association between *ADIPOQ* gene variants and obesity previously reported, although this study did not describe a causal relationship, but associations (Tables 4, 5) [5,10].

Several potential limitations of our study should be mentioned. Firstly, BMI may not be a sufficient indicator for measuring the degree of obesity. Although regional differences in body fat affect the risk of metabolic abnormalities, and although *ADIPOQ* gene variants were suggested as modulating visceral fat accumulation in some studies, other studies detected no association between *ADIPOQ* gene variants and BMI [20]. Thus, other measurements of obesity such as the waist:hip ratio or waist circumference [10,25] might be better measures of abdominal obesity in the Korean population. Secondly, there may also be some interactions with other genes related to susceptibility to obesity. For instance, evidence for an interaction between SNPs in the promoters of *ADIPOQ* (-3971A>G) and *ADIPOR1* (-3882T>C) was found [21]. Thirdly, the effects of environmental factors controlling the risk of obesity could not be evaluated in this study. Given the role of adiponectin in fatty acid oxidation, a reduction in plasma adiponectin levels may impact the ability to clear cellular adipose in obese individuals with an at-risk diplotype [20]. However, the effect of this risky polymorphism could be validly evaluated after controlling for environmental factors (e.g. physical activity, fried food intake, alcohol consumption, etc.), clinical characteristics (e.g. serum C-reactive protein, total cholesterol HDL ratio, blood pressure, etc.), and ethnicity [22,26]. Finally, the positive associations found in this study were based on the comparison made between a group of high BMI and low adiponectin subjects versus a group of low BMI and high adiponectin subjects. Thus, the statistical evidence might not reflect the effect of *ADIPOQ* gene variants on BMI and plasma adiponectin concentrations separately, but rather a joint consideration of both indices for obesity. Therefore, additional studies warrant validation of the relationship between the *ADIPOQ* gene and obesity identified in this study.

In conclusion, the SNPs identified here (i.e. -11377C>G and +45T>G) may assist in identifying subjects who are at a greater risk of obesity among healthy Koreans. Such information may provide a chance for susceptible individuals to prevent obesity and for obese individuals to determine a more efficient means of weight control. Furthermore, understanding the mechanisms by which obesity could be controlled would make it possible for us to better understand the metabolic complications of obesity.

## Figures and Tables

**Table 1:**
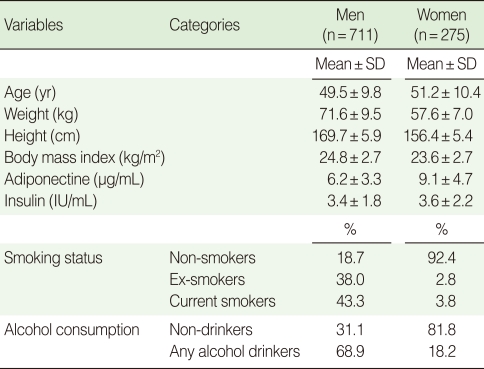
Characteristics of the study population

SD, standard deviation.

**Table 2:**
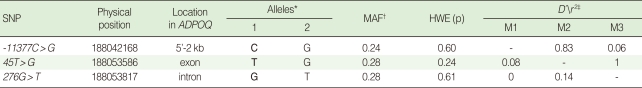
Marker information for three SNPs located in the *ADIPOQ* gene (3q27)

MAF, minor allele frequency; HWE, Hardy-Weinberg equilibrium; D: above the diagonal; *r*^2^, below the diagonal. ^*^Major alleles are in bold type; ^†^Minor allele frequencies measured among controls of G3; ^‡^Pair-wise LD was computed as both *D*' and *r*^2^.

**Table 3:**
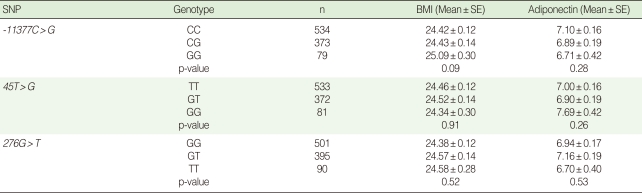
Variations in BMI (kg/mg^2^) and adiponectin concentration (µg/mL) by genotype of the three SNPs

BMI, body mass index; SE, standard error. p-values are adjusted for age and sex.

**Table 4:**
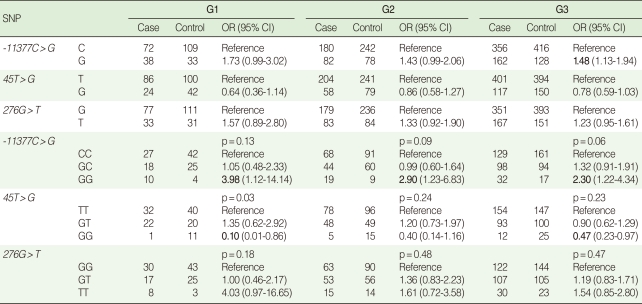
Risk estimations for alleles and genotypes of individual markers by three levels of BMI and adiponectin concentration

BMI, body mass index; OR, odds ratio; CI, confidence interval. ORs are adjusted for age and sex and 95% CI. p-values are calculated by logistic regression models.

**Table 5:**
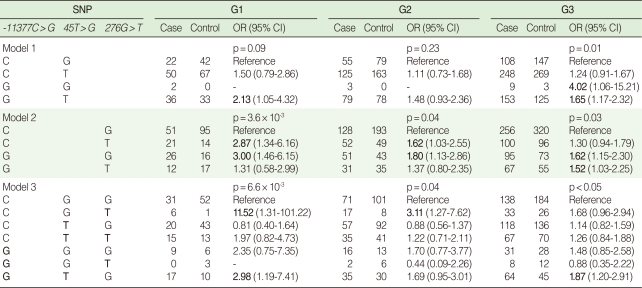
Risk estimation for 2-3 SNP haplotypes showing statistical significance by three levels of BMI and adiponectin concentration^*^

OR, odds ratio; CI, confidence interval. ORs are adjusted for age and sex. ^*^Model composed of 2 SNPs: *45T>G* and *276G>T* were not significant.
